# P090 Large vessel vasculitis with arterial and venous involvement: a diagnostic clue for a multisystem disease

**DOI:** 10.1093/rap/rkag113.120

**Published:** 2026-11-03

**Authors:** Amruth Karthik, Jagdish Nair, Vanessa Quick

**Affiliations:** Aintree University Hospital NHS Foundation Trust, Liverpool, United Kingdom; Aintree University Hospital NHS Foundation Trust, Liverpool, United Kingdom; Bedfordshire Hospitals NHS Foundation Trust, Luton, United Kingdom

## Abstract

**Introduction:**

The term large vessel vasculitis opens up a specific window of differential diagnoses. From a rheumatology perspective key clinical and demographic features may suggest differentials such as Takayasu’s arteritis and giant cell arteritis. Here we present a case of a multisystem heterogenous disease process with significant diagnostic uncertainty evolving over a period of 3 years. This complex presentation had a profound impact on patient quality of life and required a multidisciplinary approach to care.

**Case description:**

A healthy 20-year-old male presented to ophthalmology services with ocular pain and conjunctival injection. A slit lamp examination revealed bilateral acute anterior uveitis treated initially with topical steroids. Symptoms recurred a few months later with findings now in keeping with right sided scleritis. In the coming months he developed features in keeping with an inflammatory seronegative arthritis, alongside lower back pain. His MRI spine spondyloarthritis protocol showed no features concerning for an axial spondyloarthropathy. Methotrexate was efficacious for his joint symptoms, however episodic scleritis and episcleritis persisted.

Subsequently, he presented acutely with pleuritic chest pain at 7 and 13 months post initial symptom onset. After his second presentation a diagnosis of pericarditis was made. An echocardiogram revealed a pericardial effusion and a cardiac MRI confirmed this with increased pericardial enhancement. Colchicine was initiated alongside a tapering corticosteroid regime. Immunology, virology and bacteriology were unremarkable including a normal HLA-B51.

2 months later given constitutional symptoms a PET-CT scan was arranged unmasking a pan-aortitis. This was treated with high dose corticosteroids and subsequently augmented with tocilizumab therapy. Following travel to Japan 6 months later he developed thromboses in his left subclavian vein, right popliteal vein and femoral vein; initially treated with rivaroxaban. Episodic abdominal pain accompanied this and a MRA scan highlighted occlusion of both coeliac arteries and superior mesenteric artery. At this point a unifying diagnosis of Behçet’s syndrome was reached after further history revealed longstanding oral aphthous ulceration and acneiform lesions. No history of pathergy was noted. Tocilizumab and methotrexate were substituted with azathioprine and infliximab resulting in no further vascular events. Clinical psychology support was offered via the specialist multidisciplinary Behçet’s service given the profound impact of 3 years of disease on quality of life.

**Discussion:**

This case demonstrates a sequential unmasking of Behçet’s syndrome complicated with stenotic large vessel vasculitis. History taking remains paramount. Crucial information regarding longstanding colchicine responsive oral aphthous ulceration was unearthed later after referrals to different rheumatology services. A history of acneiform lesions were also uncovered. Episodic pericarditis was an additional feature that complicated this case, and is described in the literature as the commonest cardiovascular manifestation of Behçet’s syndrome.

The PET- CT scan formed a cornerstone of the diagnostic process. Markedly increased tracer uptake with pan-aortitis confirmed large vessel vasculitis, and warranted a review of immunosuppression. The atypia of stenotic predominance of the vascular lesions over aneurysm seen on the MRA scan further posed a challenge to reach a unifying diagnosis. A differential of Takayasu’s arteritis in the context of large vessel vasculitis in a young patient was initially considered. Hence, tocilizumab was decided as the initial biologic drug of choice. Monogenic diseases were also considered but no clear pattern was identified. IL-6 inhibition was not successful in controlling disease progression and the patient developed a significant burden of venous thrombosis.

Anticoagulation in venous thrombotic disease associated with Behçet’s syndrome is not routinely recommended and if considered arterial aneurysm needs to be excluded. In this case Infliximab alongside azathioprine were employed effectively for the widespread vascular disease. This is in concordance with the now established 2025 British Society of Rheumatology Behçet’s guidelines. Unfortunately, this case also effectively highlights the management challenges and poor prognostic features of vascular Behçet’s with low dose prednisolone still required to control disease activity. This patient had setbacks throughout his disease evolution with repeated hospital admissions alongside hinderance of career progression. Psychology support was imperative to address this as part our specialist multidisciplinary service to allow the ability to process the disease impact.

**Key learning points:**

• Behçet’s syndrome can cause inflammatory disease throughout the entire vascular tree; large vessel vasculitis can manifest with aneurysmal or stenotic disease requiring aggressive immunosuppression.

• In depth history taking searching for classical mucocutaneous features is integral to diagnosis.

• Disease evolution can be episodic or insidious with widespread manifestations including pericarditis; this often can cause diagnostic challenge.

• Non-specific symptoms such as weight loss, fatigue and pyrexia require appropriate investigation with consideration of PET CT imaging. Abdominal pain may indicate arterial insufficiency in the correct context.

• Multi-disciplinary team management is essential; specialist Behçet’s services form a key role in assisting in local management particularly with psychology support for impact on mental health.

